# Supplementary material for: Fasudil Increased the Sensitivity to Gefitinib in NSCLC by Decreasing Intracellular Lipid Accumulation
Source: Cancers (Basel). 2022 Sep 27;14(19):4709. doi: 10.3390/cancers14194709 (PMC9563767; doi:10.3390/cancers14194709)
Supplement: Supplementary file 1 [file cancers-14-04709-s001.zip › Supplementary File S1.pdf]

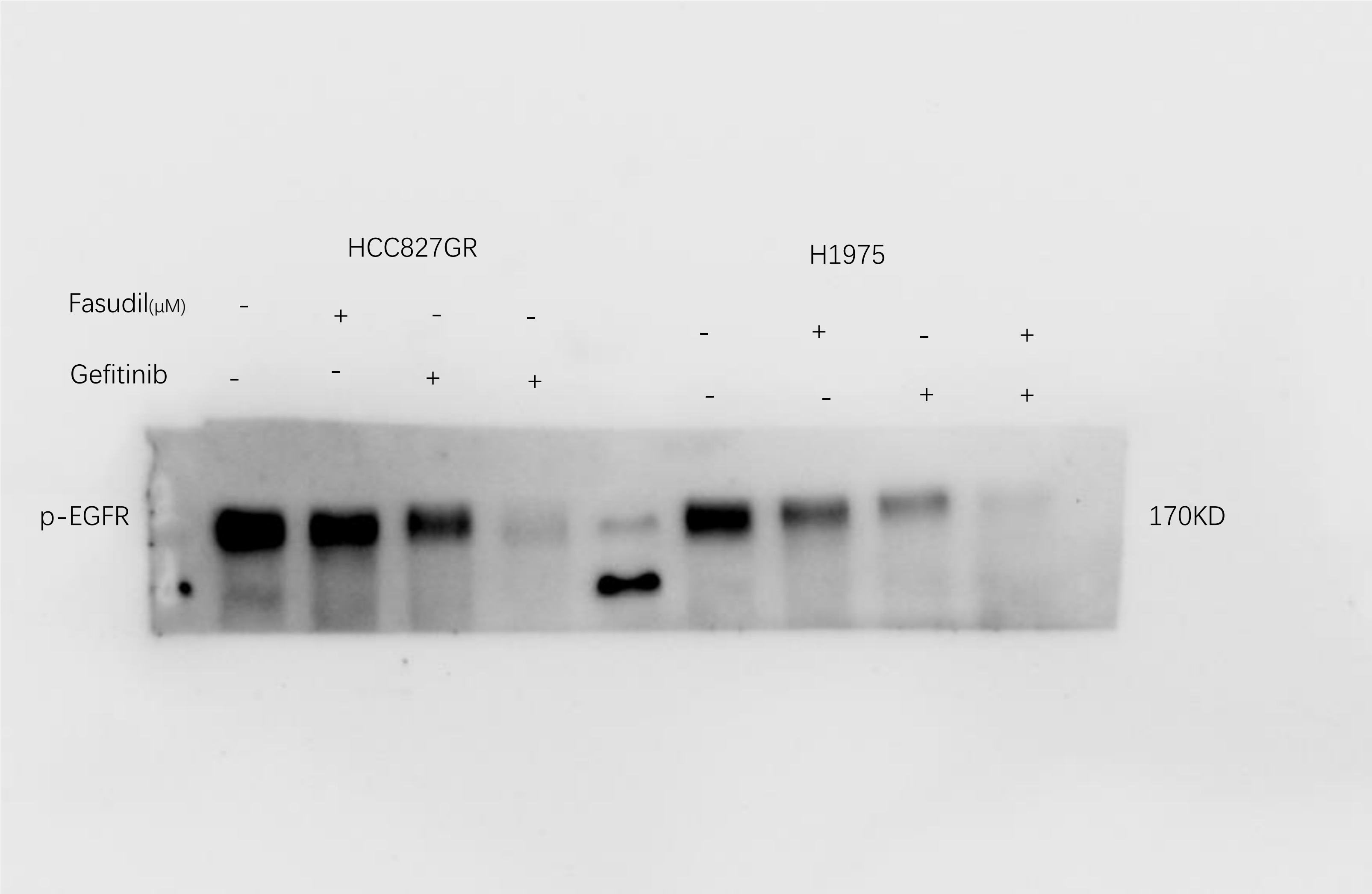

Figure 2G

Figure 2G

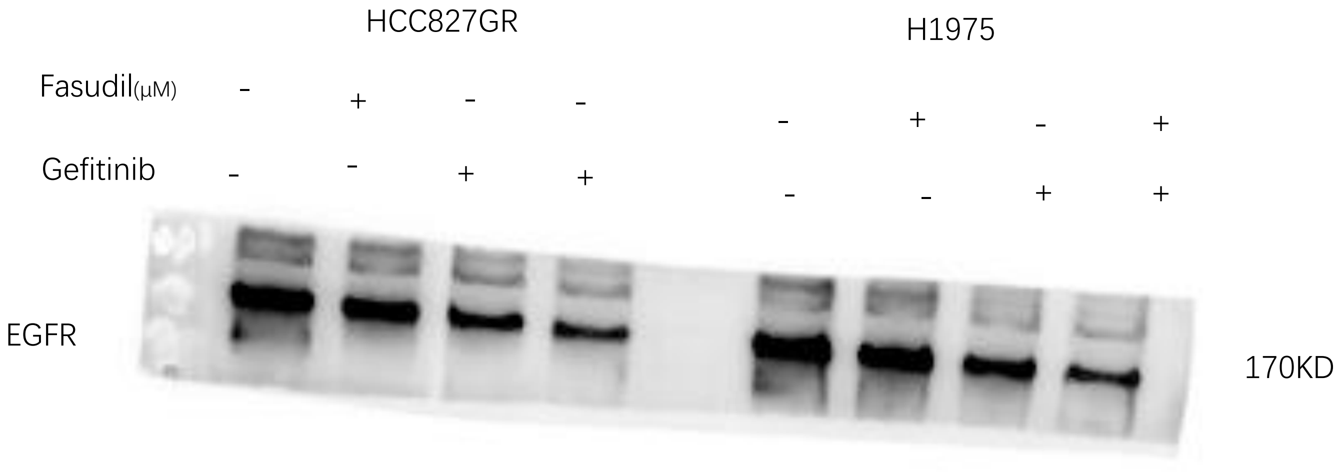

Figure 2G

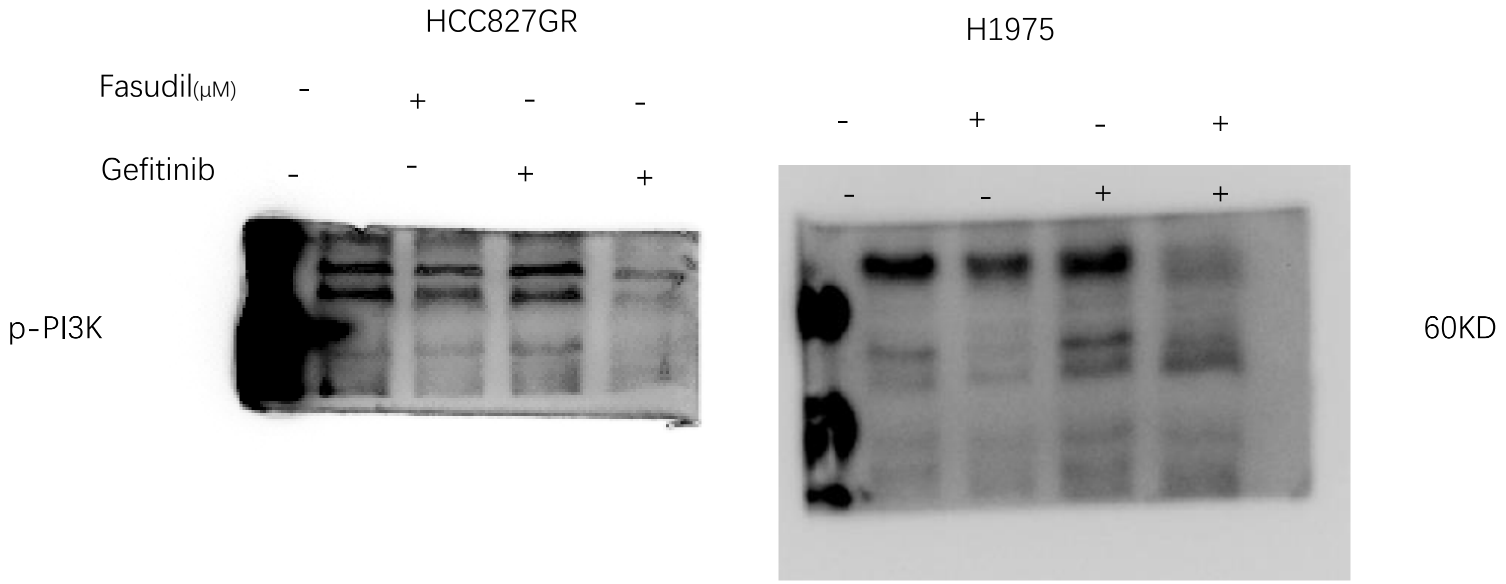

Figure 2G

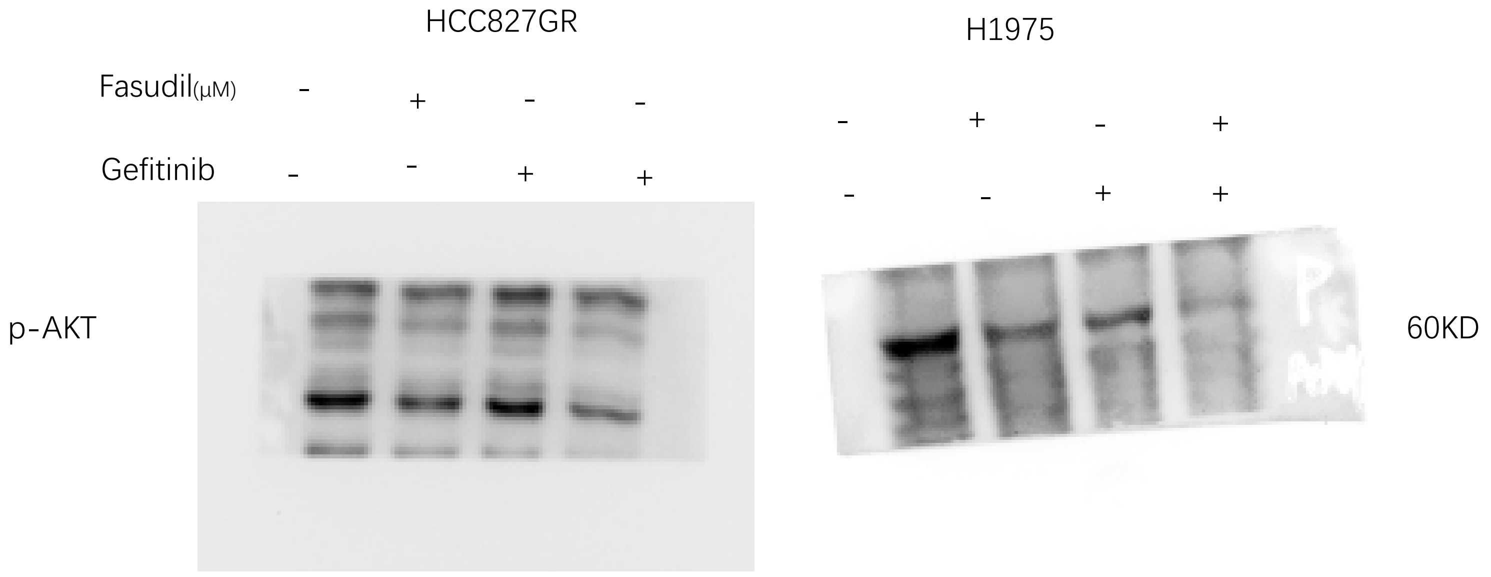

Figure 2G

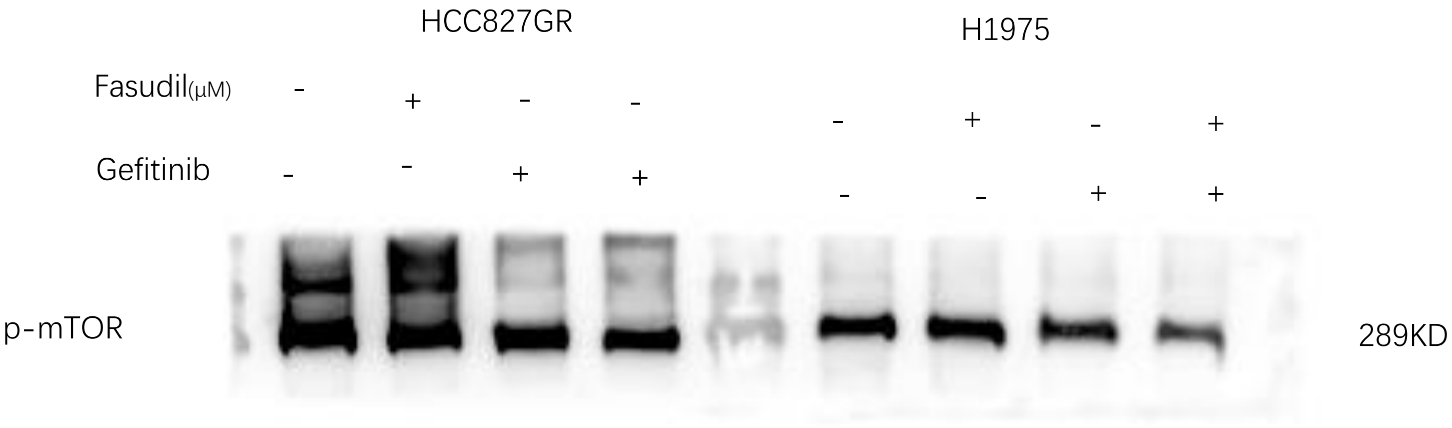

Figure 2G

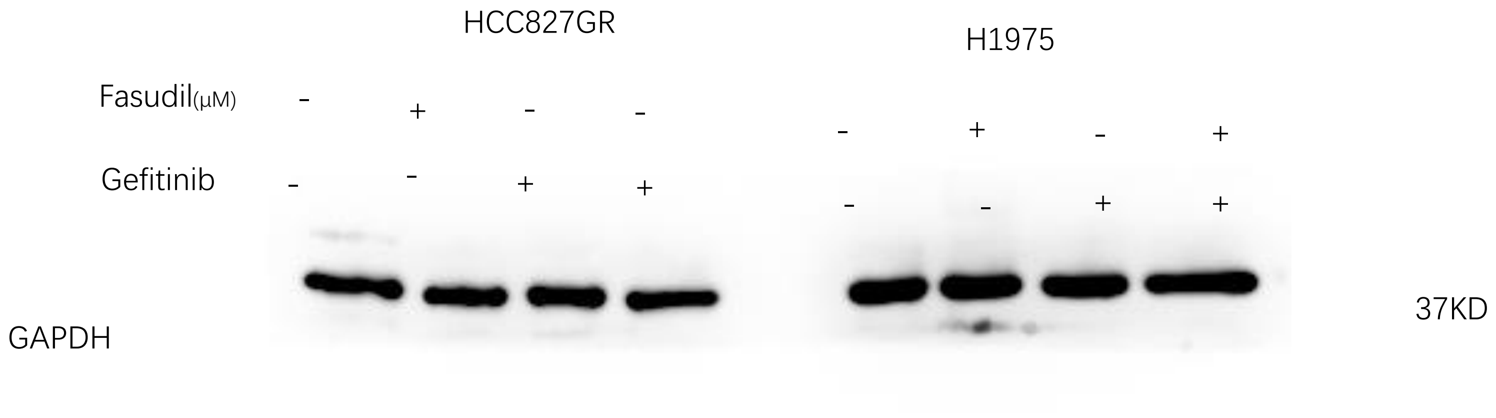

Figure 3F

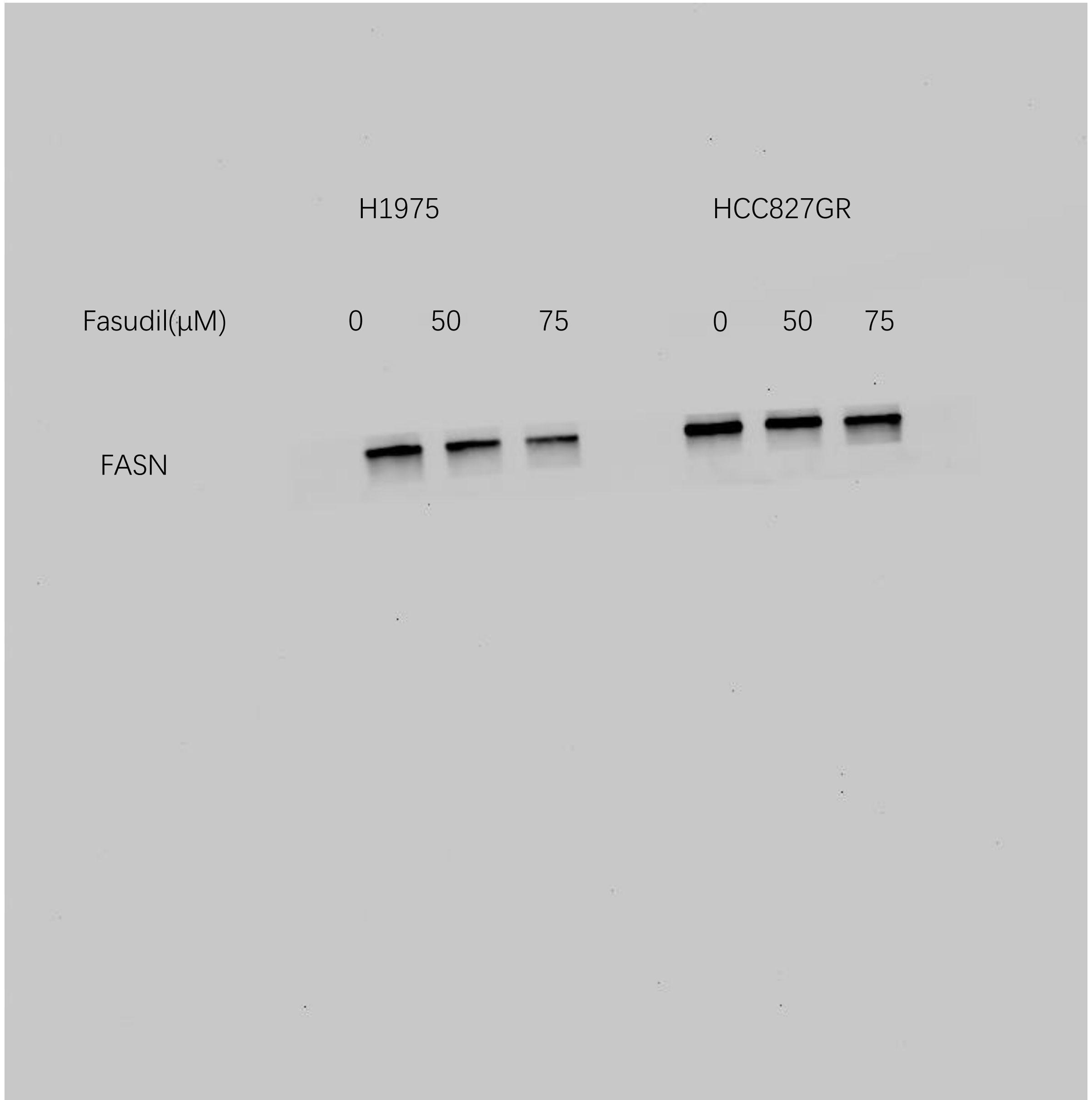

273KD

Figure 3F

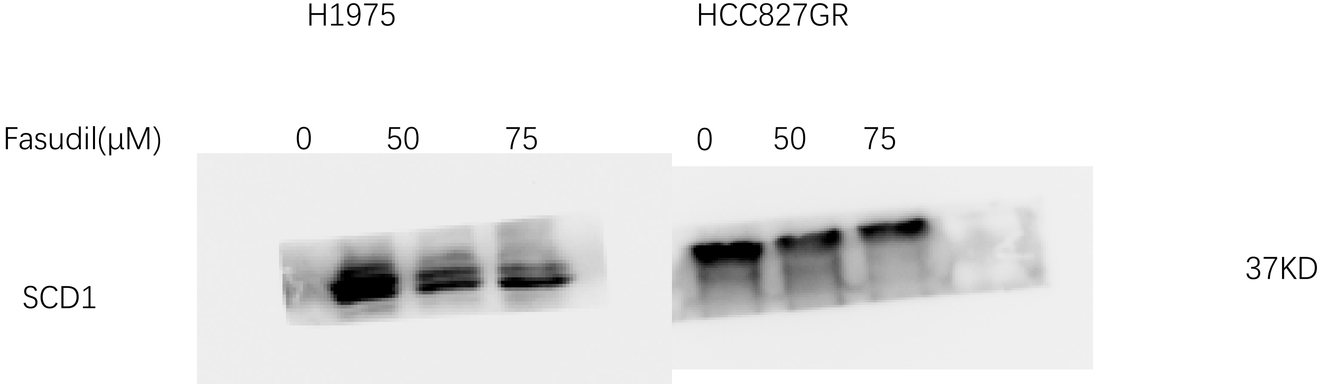

Figure 3F

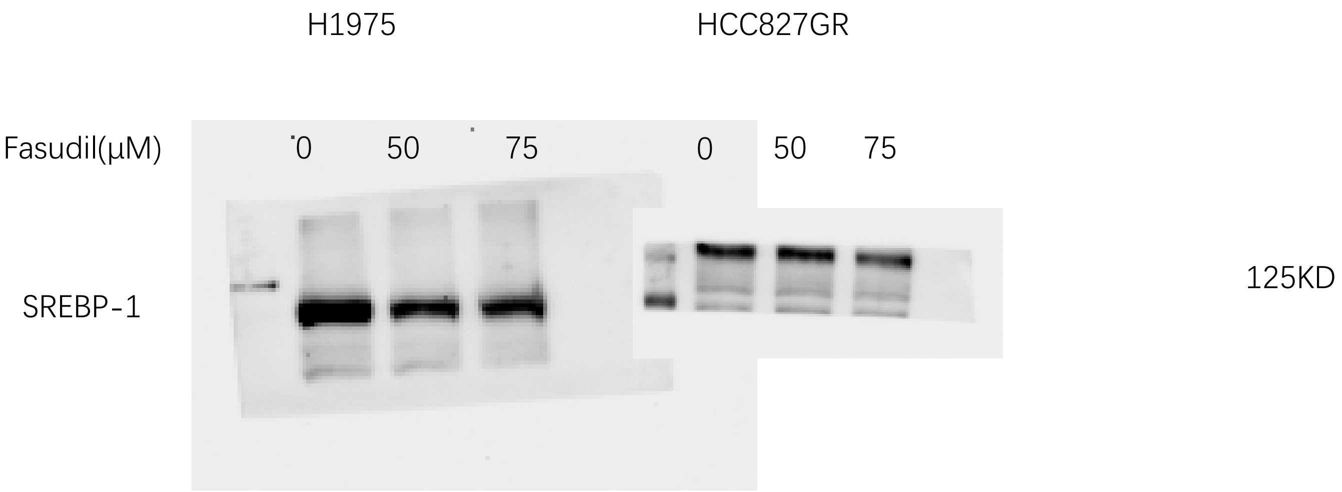

Figure 3F

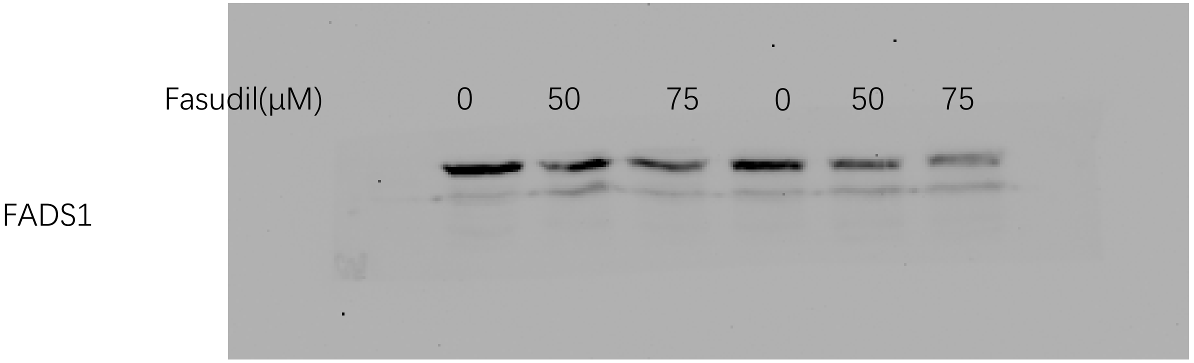

Figure 3F

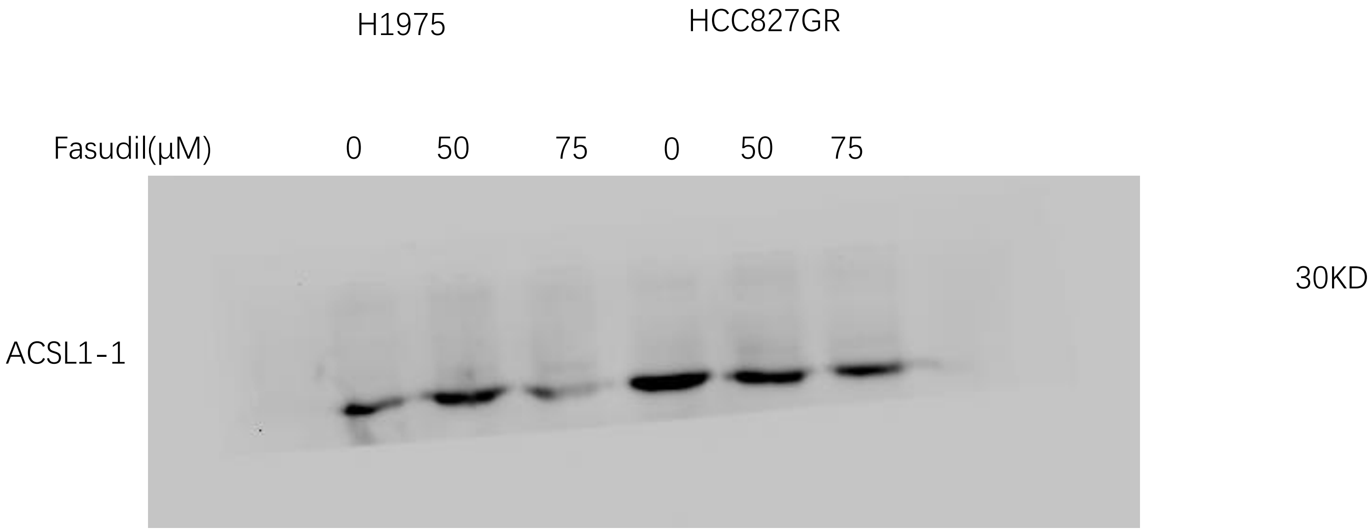

Figure 3F

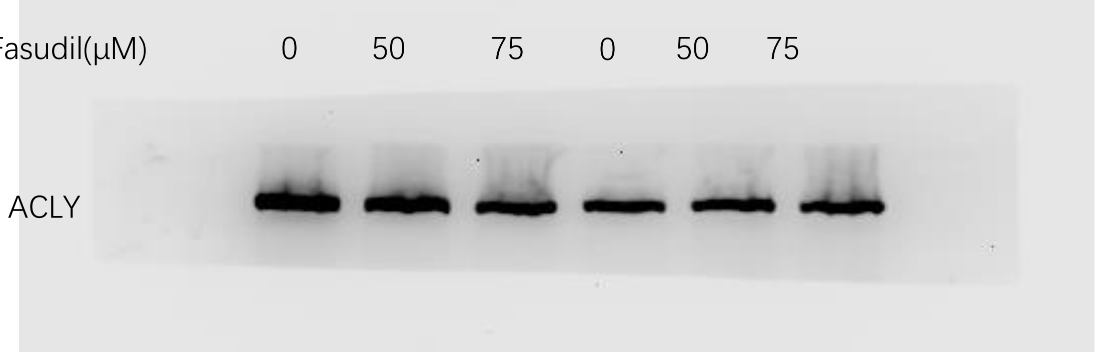

Figure 3F

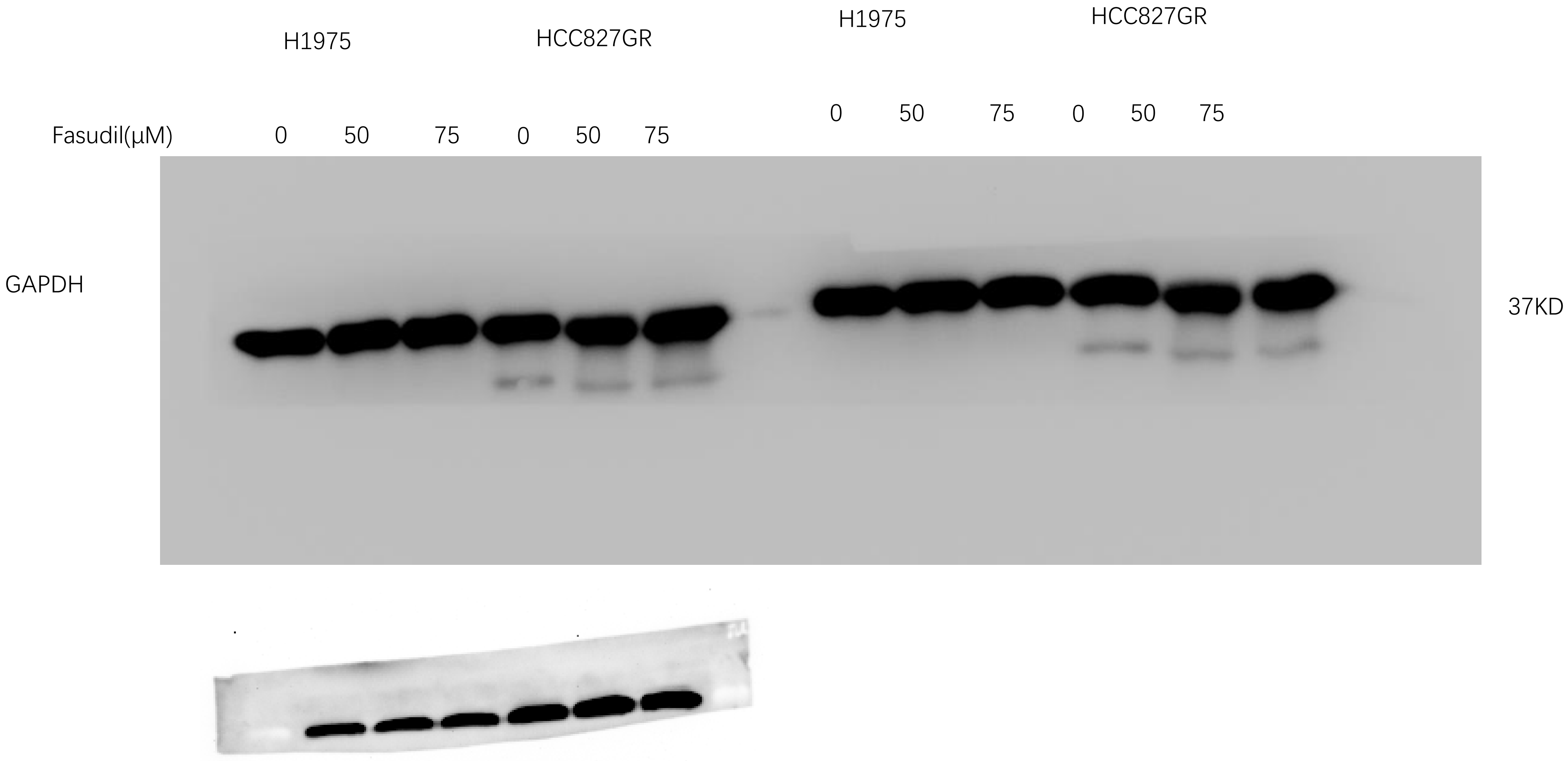
H1975
HCC827GR
H1975
HCC827GR
Fasudil(μM)
0 50 75 0 50 75
0 50 75 0 50 75
GAPDH
37KD

Figure 3F

H1975 HCC827GR

Fasudil(μM) 0 50 75 0 50 75

GAPDH

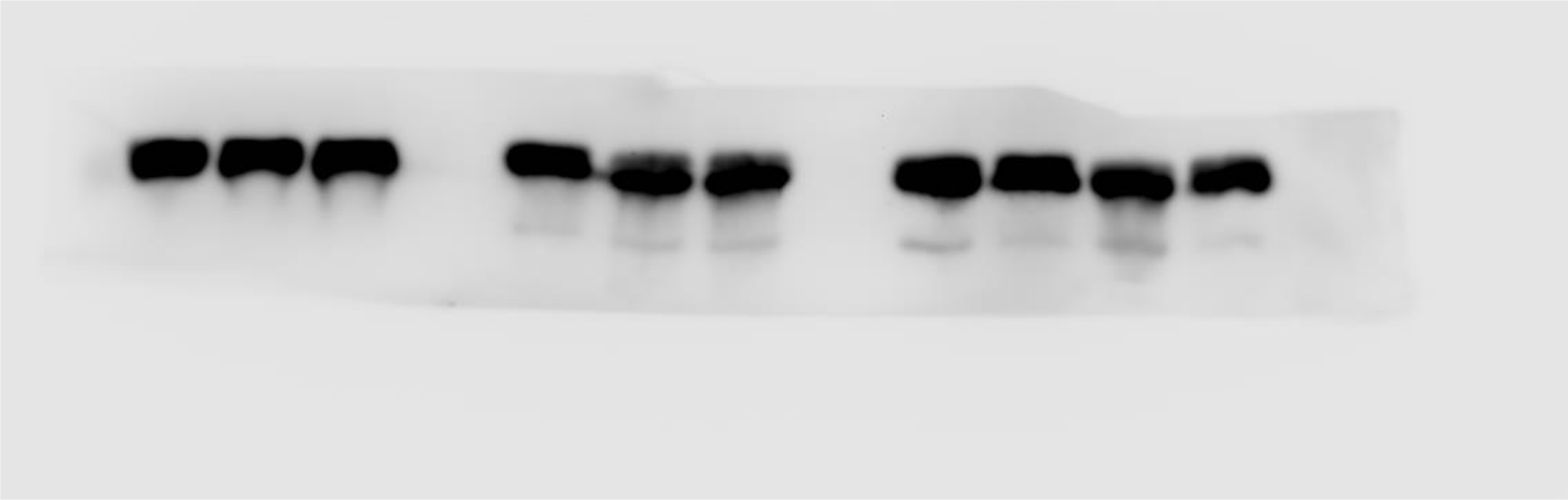

37KD

Figure 4B

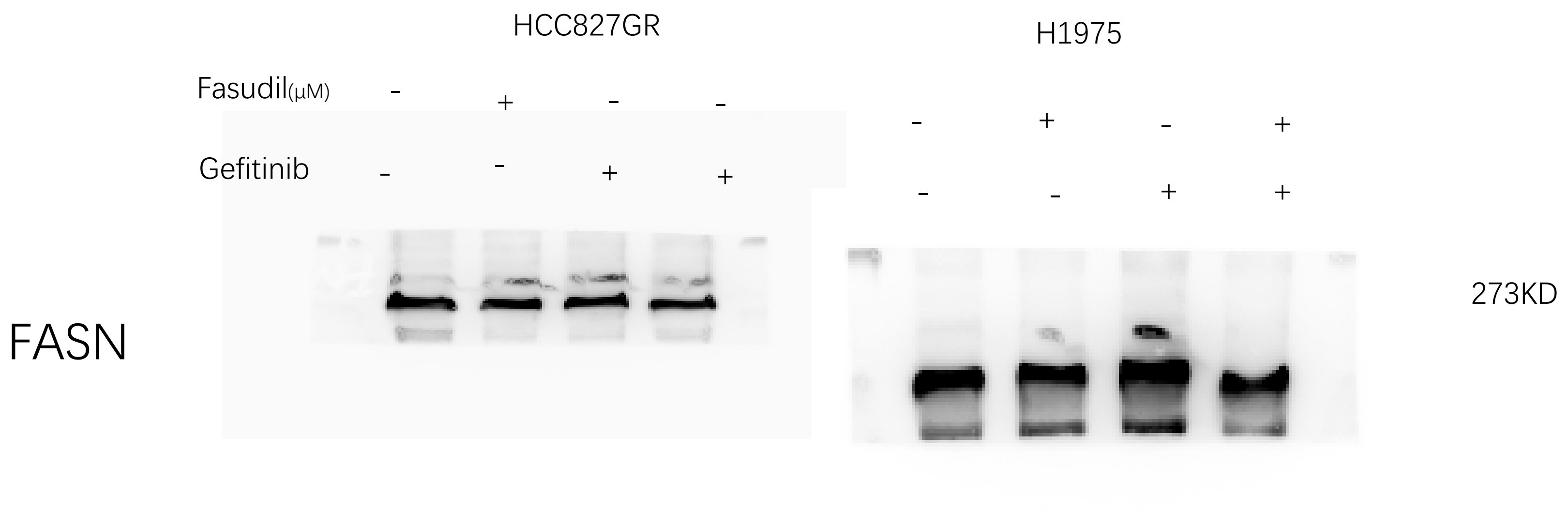

Figure 4B

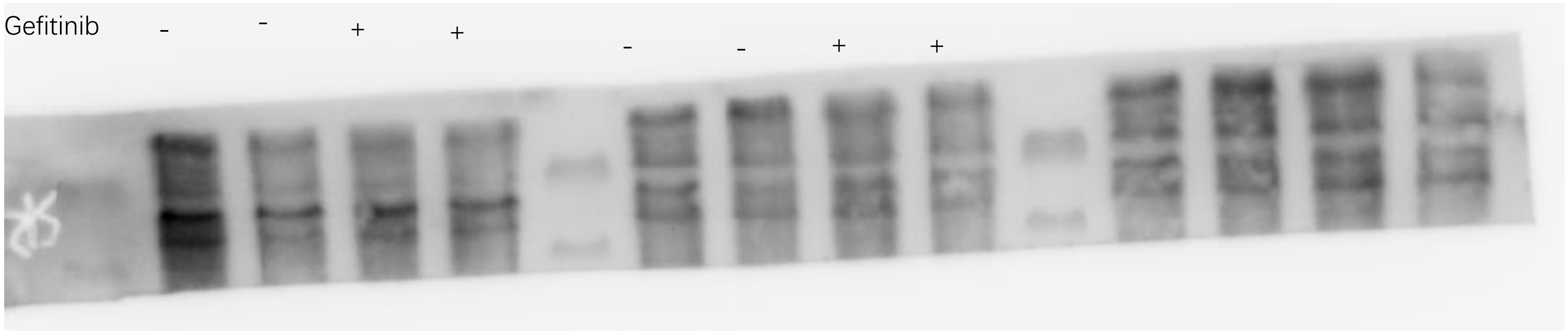

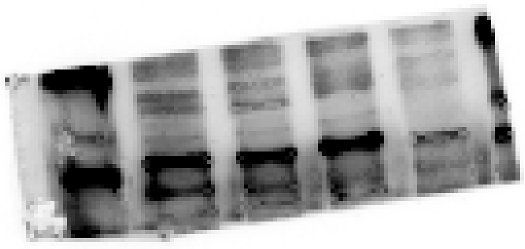

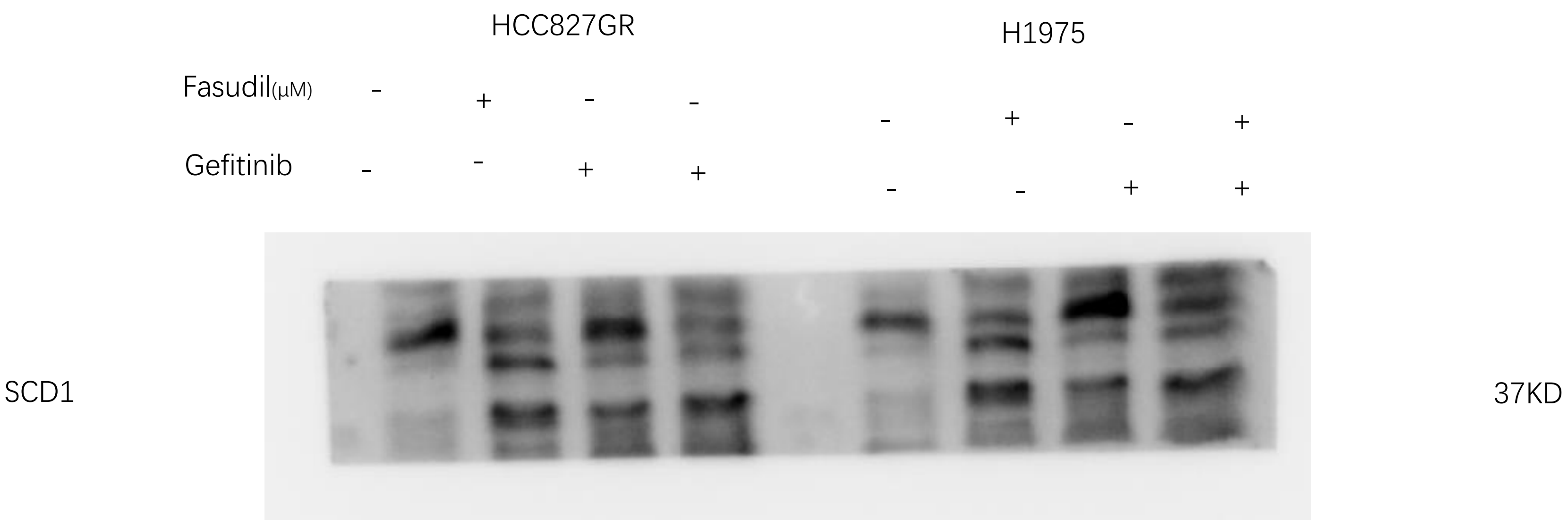

Figure 4B

Figure 4B

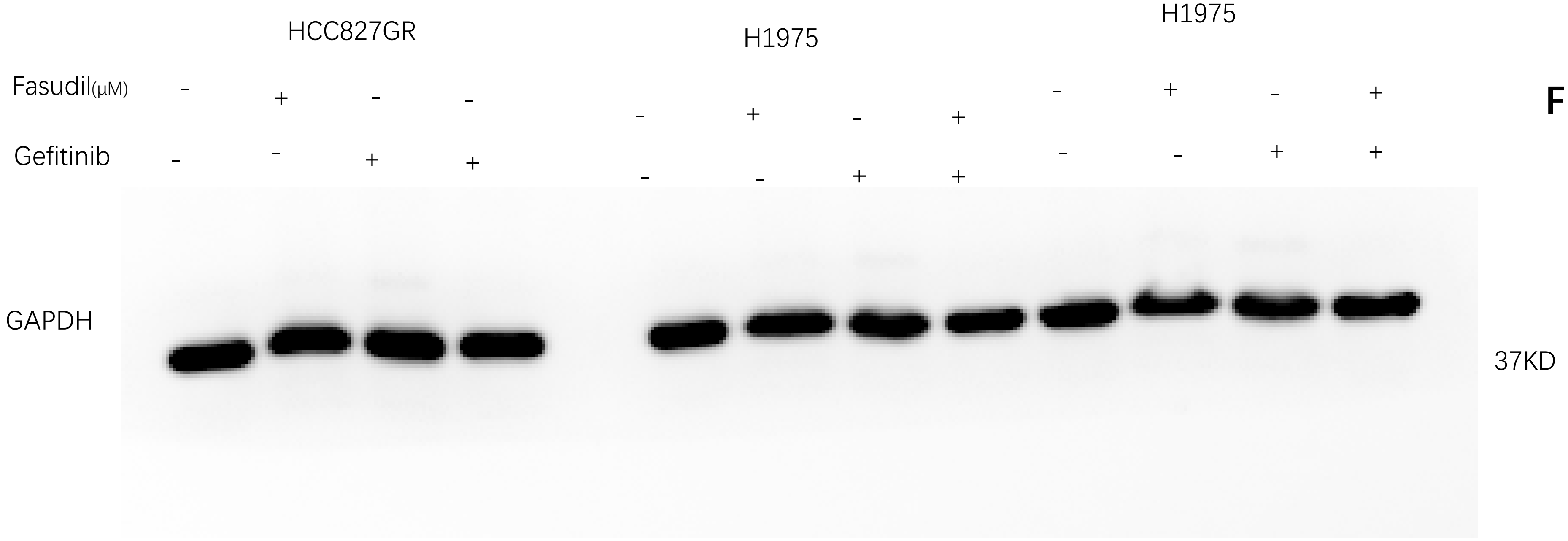

Figure 5A

H1975 HCC827GR

Fasudil(μM) 0 50 75 0 50 75

62KD

AMPK

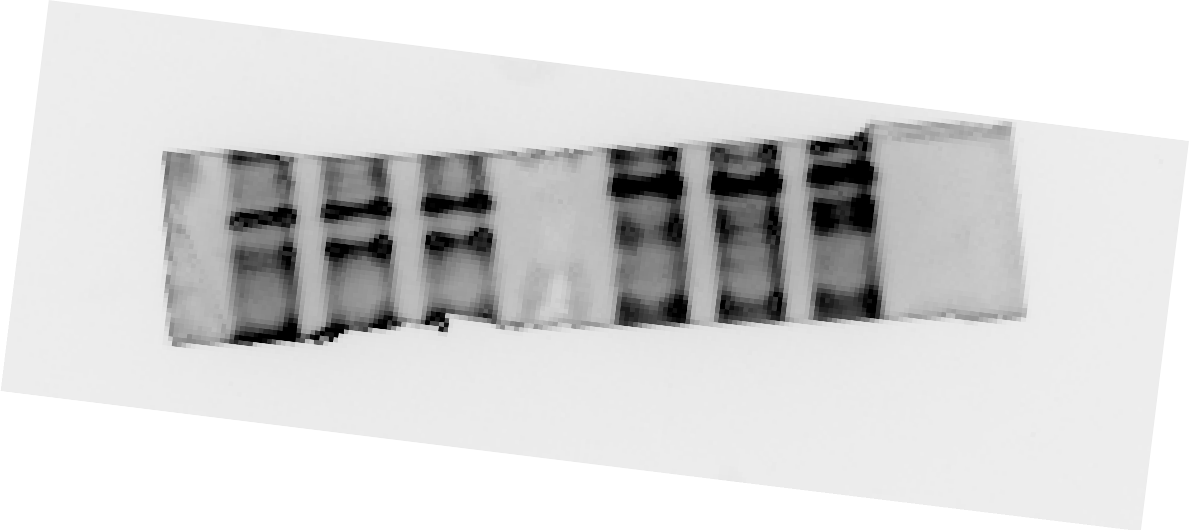

Figure 5A

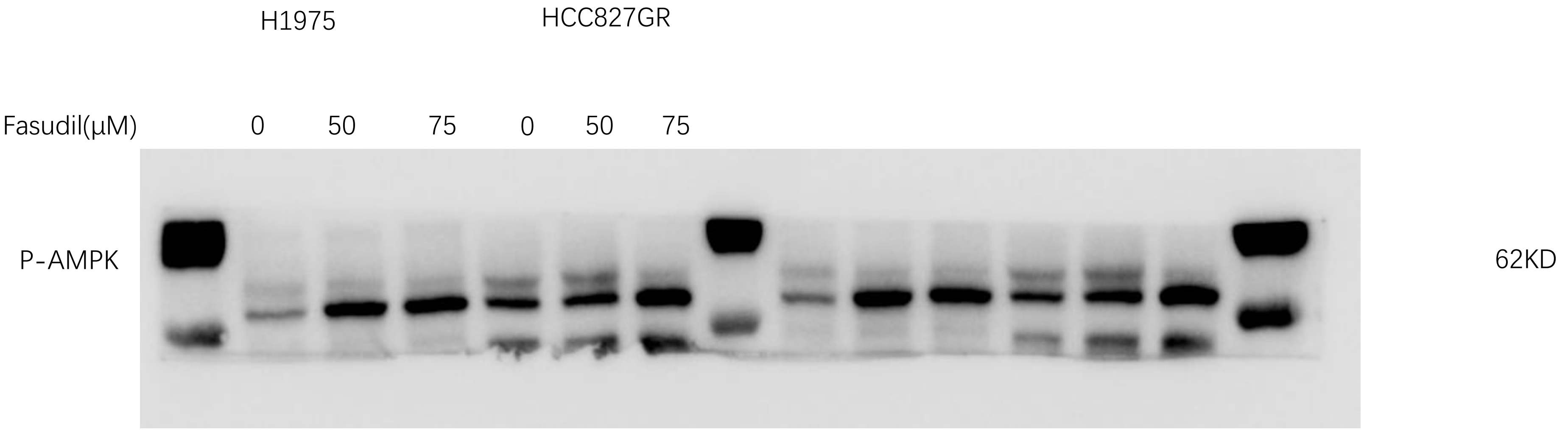

Figure 5A

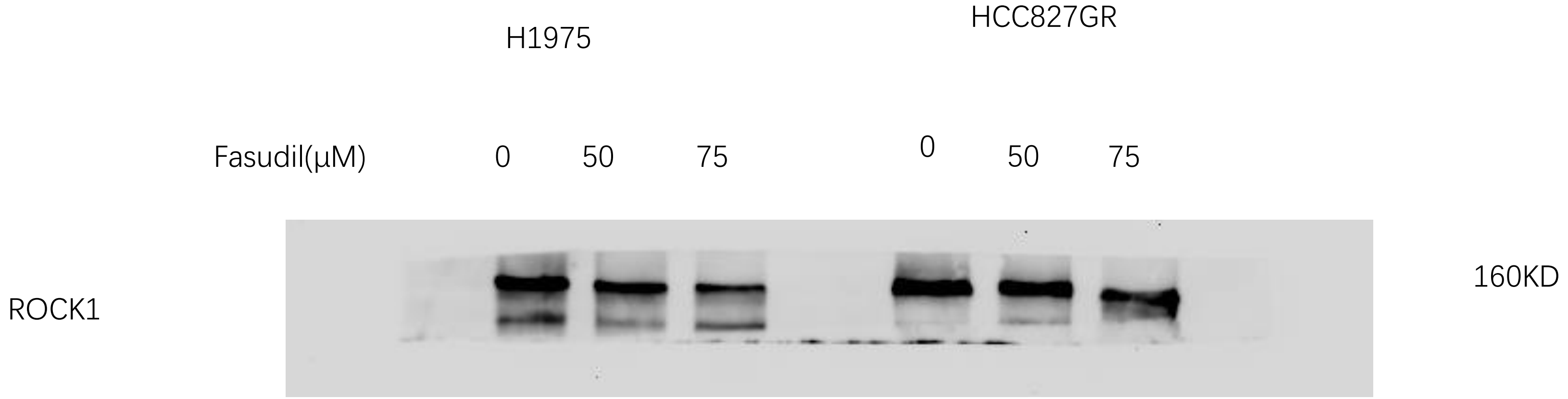

Figure 5A

H1975 HCC827GR

Fasudil(μM) 0 50 75 0 50 75

GAPDH

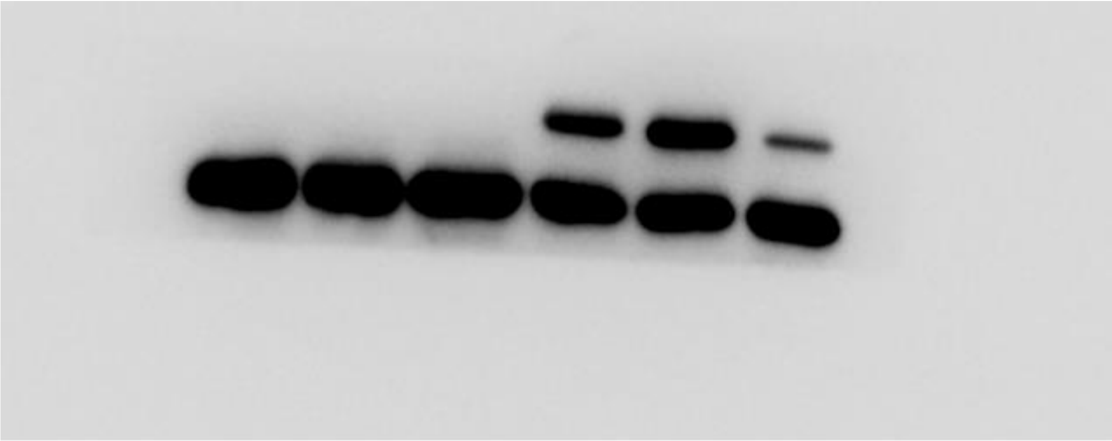

H1975 HCC827GR

Fasudil(μM) 0 50 75 0 50 75

GAPDH

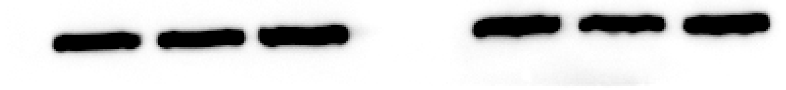

Figure 5B

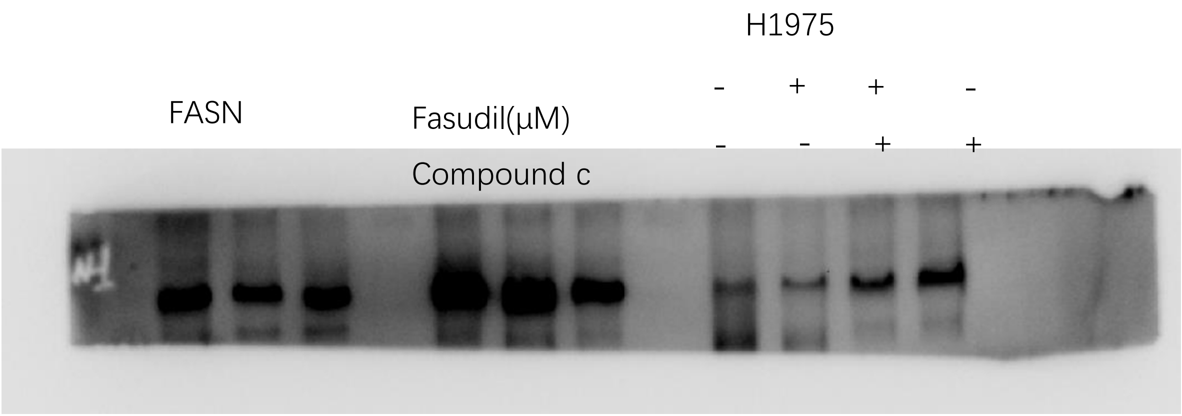

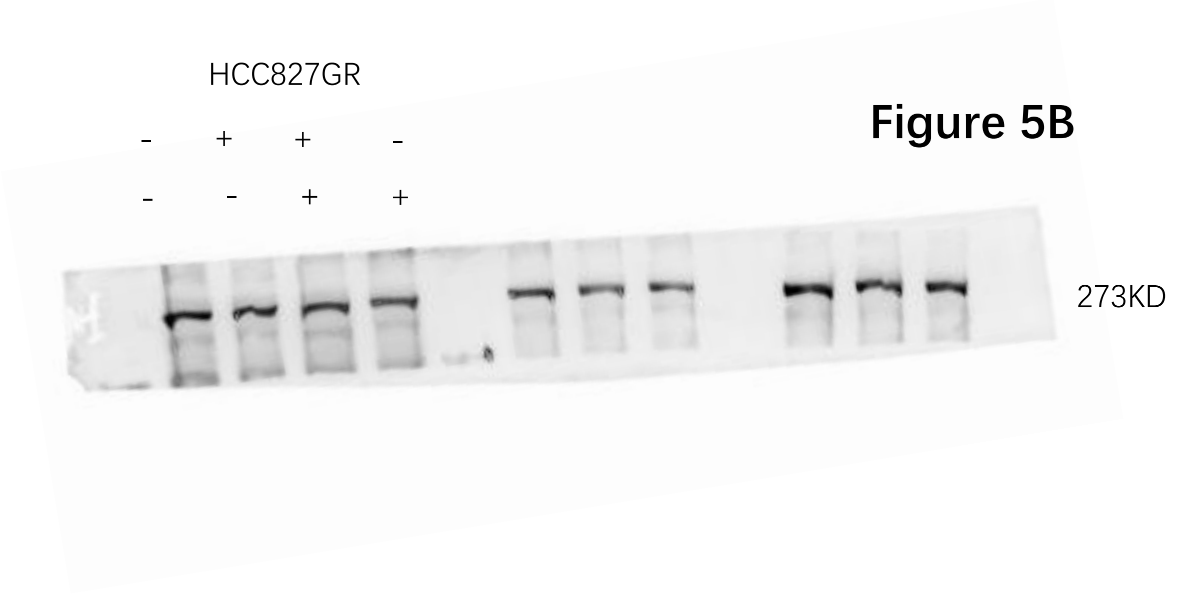

Figure 5B

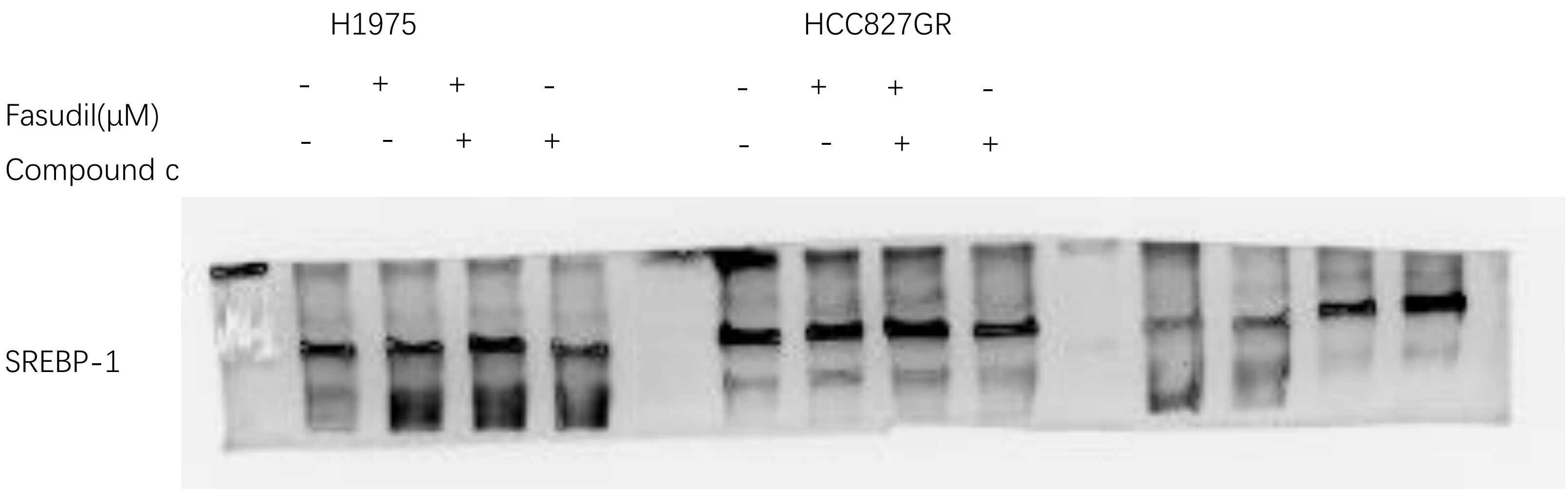

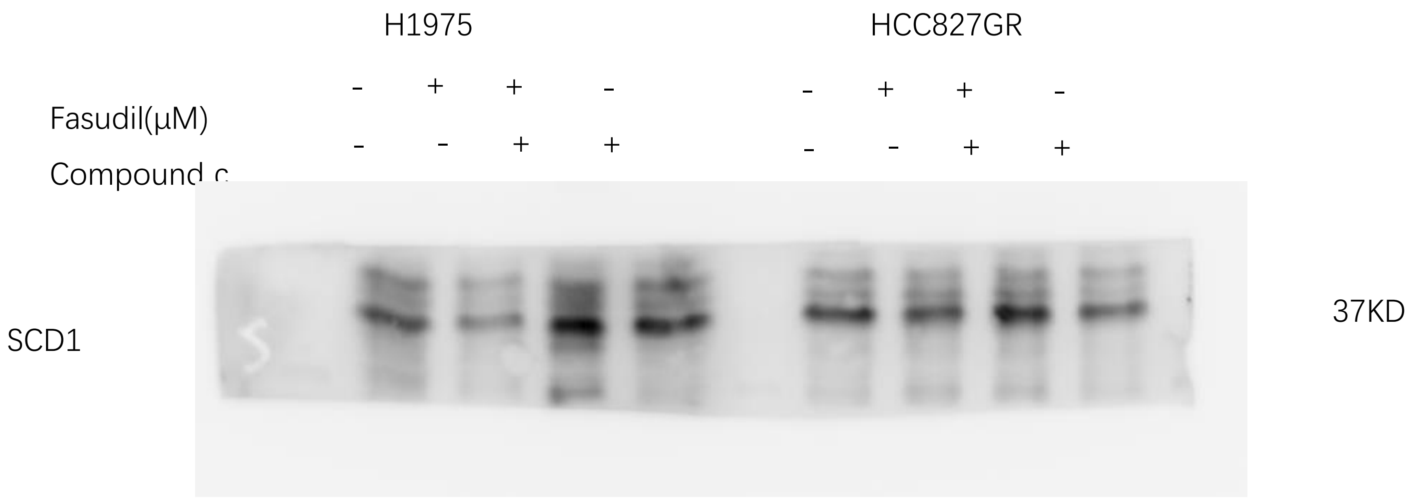

Figure 5B

Figure 5B

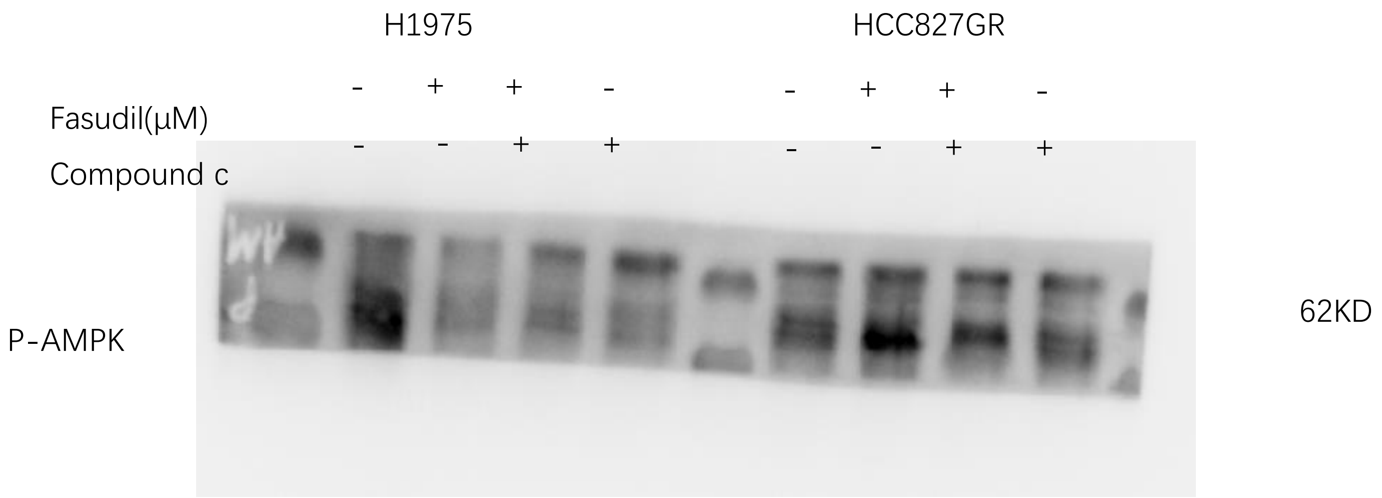

Figure 5B

H1975 HCC827GR

- + + - - + + -

Fasudil(μM)

- - + + - - + +

Compound c

GAPDH

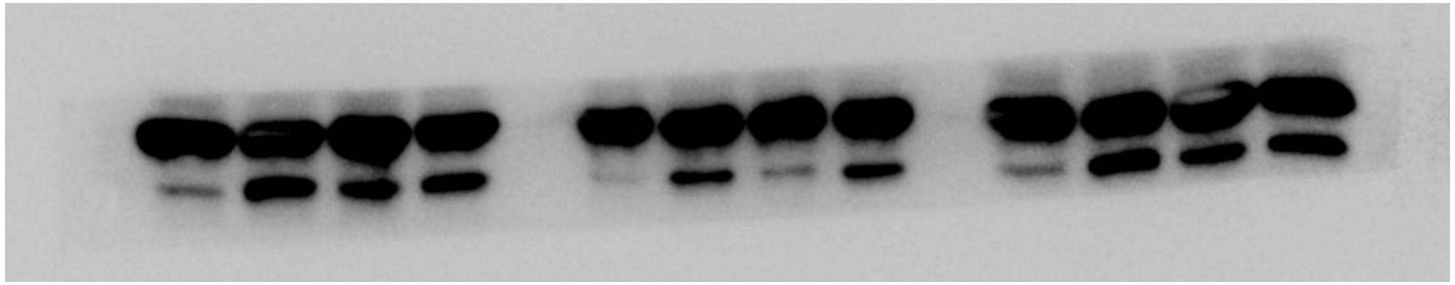

37KD
