# Supplementary material for: Fasudil Increased the Sensitivity to Gefitinib in NSCLC by Decreasing Intracellular Lipid Accumulation
Source: Cancers (Basel). 2022 Sep 27;14(19):4709. doi: 10.3390/cancers14194709 (PMC9563767; doi:10.3390/cancers14194709)
Supplement: Supplementary file 1 [file cancers-14-04709-s001.zip › Supplementary materials.pdf]

Fasudil Increased the Sensitivity to Gefitinib in NSCLC by Decreasing Intracellular Lipid Accumulation

Tingting Liao [1,2,†], Jingjing Deng [1,†], Wenjuan Chen [1], Juanjuan Xu [1], Guanghai Yang [3], Mei Zhou [1], Zhilei Lv [1], Sufei Wang [1], Siwei Song [1], Xueyun Tan [1], Zhengrong Yin [1], Yumei Li [1] and Yang Jin [1,*]

Supplementary Materials

Supplementary Figure S1

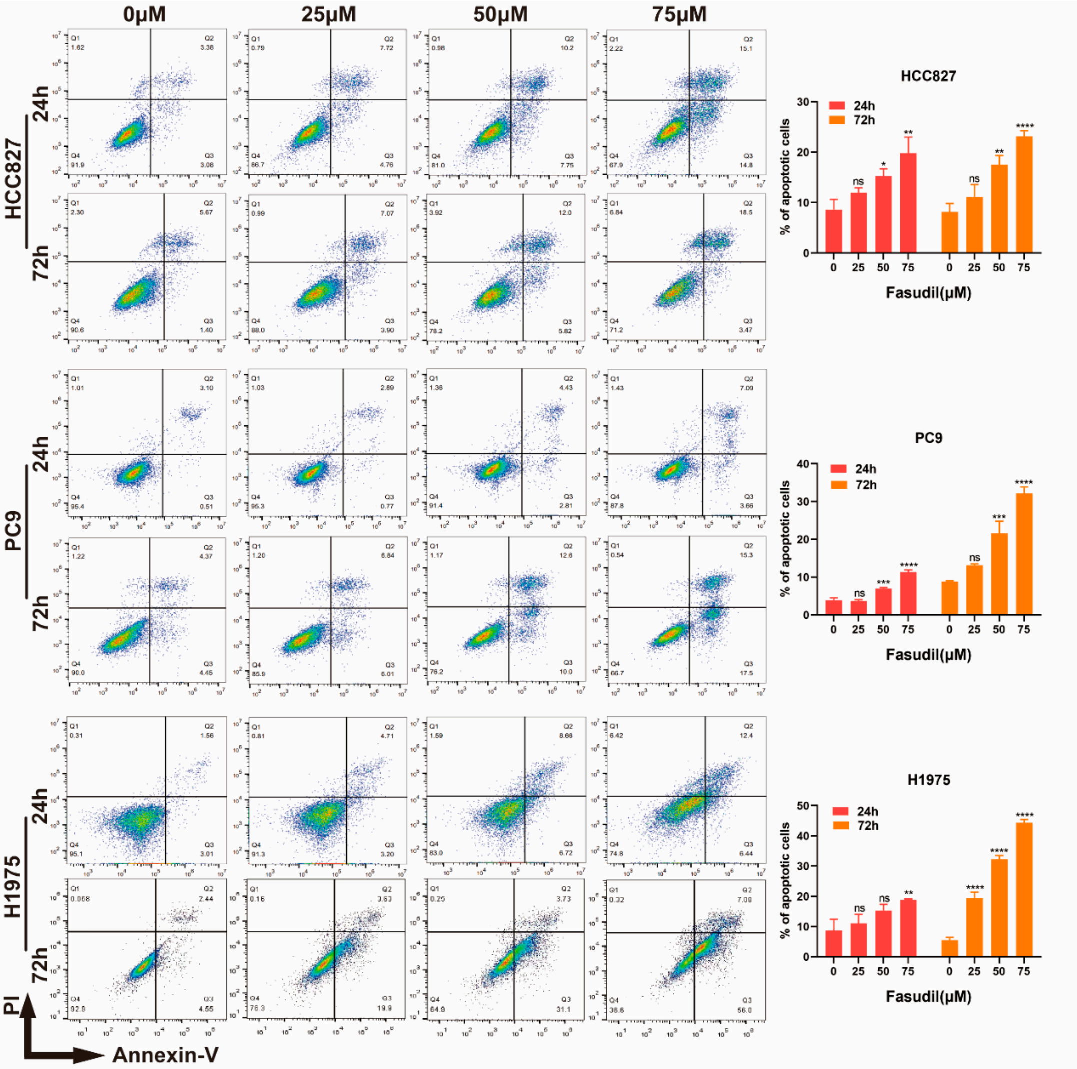

Figure S1. Apoptosis analysis of EGFR-mutation NSCLC cells in vitro by Fasudil. HCC827, PC9 and H1975 had been treated with 25μM, 50μM and 75μM Fasudil for 24h and 72h separately, and the cells treated with ddH$_2$O were served as the control group. Afterwards the cells were stained with Annexin V-FITC/PI. Subsequently percentages of apoptotic cells (Annexin-V$^+$PI$^-$ + Annexin-V$^+$PI$^+$) were analyzed via flow cytometry. ns: no statistical significance, * $P < 0.05$, ** $P < 0.01$, *** $P < 0.001$, **** $P < 0.0001$.

Supplementary Figure S2

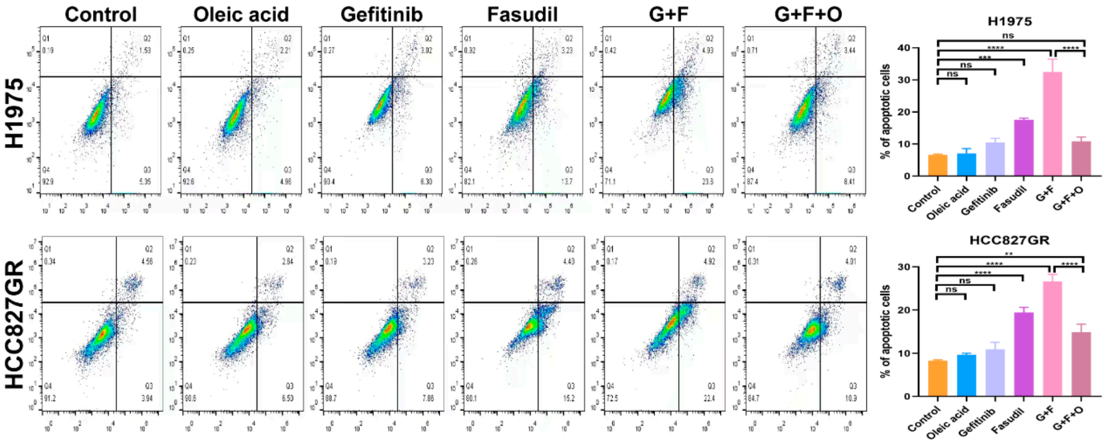

Figure S2. Oleic acid rescue experiment of H1975 and HCC827GR cell lines. Both H1975 and HCC827GR cells were treated with Control (ddH2O), Oleic acid (2%), Fasudil (50 μM), Gefitinib (15 μM), Fasudil (50 μM, F) + Gefitinib (15 μM, G) or Fasudil (50 μM, F) + Gefitinib (15 μM, G) + Oleic acid (20×, O) for 48h, and subsequently the apoptosis assay was conducted. ns: no statistical significance, ** $P < 0.01$, *** $P < 0.001$, **** $P < 0.0001$.
